# Protective Effect of Isoorientin on Oleic Acid-Induced Oxidative Damage and Steatosis in Rat Liver Cells

**DOI:** 10.3389/fphar.2022.818159

**Published:** 2022-02-03

**Authors:** Tongwang Luo, Sheng Jiang, Bin Zhou, Quanjiang Song, Jing Du, Ping Liu, Xiaodu Wang, Houhui Song, Chunyan Shao

**Affiliations:** ^1^ Key Laboratory of Applied Technology on Green-Eco-Healthy Animal Husbandry of Zhejiang Province, Hangzhou, China; ^2^ Zhejiang Provincial Engineering Laboratory for Animal Health Inspection and Internet Technology, Hangzhou, China; ^3^ Zhejiang International Science and Technology Cooperation Base for Veterinary Medicine and Health Management, Hangzhou, China; ^4^ China-Australia Joint Laboratory for Animal Health Big Data Analytics, Hangzhou, China; ^5^ College of Animal Science and Technology, College of Veterinary Medicine, Zhejiang A&F University, Hangzhou, China

**Keywords:** isoorientin, oleic acid, steatosis, oxidative damage, PPARγ, NF-kB p65

## Abstract

The harm of nonalcoholic fatty liver disease to human health is increasing, which calls for urgent prevention and treatment of the disease. Isoorientin is an effective ingredient of Chinese herbal medicine with anti-inflammatory and antioxidant effects. However, the effect of isoorientin in nonalcoholic fatty liver disease is still unclear. In this study, combined *in vivo* and *in vitro* experiments, through pathological observation, flow cytometry, immunofluorescence and western blot analysis to explore the role of isoorientin in steatosis and reveal its molecular mechanism. The results demonstrated that oleic acid treatment significantly increased the content of ROS and lipid droplets in rat hepatocytes, and promoted the expression of γH2AX, HO-1, PPARγ, SREBP-1c, FAS. The ROS content in the cells of co-treated with isoorientin and oleic acid was significantly reduced compared to the oleic acid group, and the expression of γH2AX, HO-1, PPARγ, SREBP-1c, FAS, and the nuclear translocation of NF-κB p65 were also significantly inhibited. Our data showed that oleic acid induce oxidative damage and steatosis in hepatocytes both *in vitro* and *in vivo*, and activate the PPARγ/NF-κB p65 signal pathway. Moreover, isoorientin can significantly reduce oleic acid -induced oxidative damage and steatosis by regulating the PPARγ/NF-kB p65 signal pathway.

## Introduction

Fatty liver disease refers to excessive accumulation of fat in liver cells, which is a serious threat to people’s health. Fatty liver can be divided into two categories: alcoholic fatty liver and non-alcoholic fatty liver. Previous studies have reported that 10–20% of nonalcoholic fatty liver will develop into nonalcoholic steatohepatitis, and about 30–50% of nonalcoholic steatohepatitis patients will further develop liver fibrosis, cirrhosis, and even liver cancer within 10 years (Zoller and Tilg, 2016; Loomba et al., 2021). The incidence of nonalcoholic fatty liver disease among patients with liver diseases in China is second only to viral hepatitis, and the harm to human health is increasing. Therefore, there is an urgent need for prevention and treatment of the disease.

Oleic acid (OA) is the most abundant monounsaturated free fatty acid in the serum of nonalcoholic fatty liver. It exists in animals and plants, and is an indispensable nutrient in animal food. In addition, it plays a very important role in the occurrence and development of fatty liver (Jiao et al., 2020; Li L. et al., 2020). Although OA is an indispensable nutrient in animal food, excessive amounts can also cause cell damage. Studies have shown that OA can enhance oxidative damage in rat brain, and induce oxidative stress in L02 and HepG2 cells (Yu et al., 2018). Our previous study have also showed that OA can cause oxidative stress and induce steatosis in BRL-3A cells (Zhang et al., 2019). However, its specific molecular mechanism has not yet been elucidated.

Peroxisome proliferator-activated receptors (PPARs) are members of the nuclear receptor superfamily that modulate various metabolic processes, such as energy expenditure, inflammatory response, and lipid metabolism (Hong et al., 2019). There are three subtypes of PPAR: PPARα, PPARδ, and PPARγ (Hong et al., 2021). PPARγ is widely expressed in lung tissue, adipose tissue, and immune cells, and is closely associated with the metabolism of fatty liver. PPARγ can protect neurons and glial cells from oxidative damage, mitochondrial dysfunction, and apoptosis (Hacioglu et al., 2021). Besides, PPARγ plays an important role in the regulation of nuclear factor-kappaB (NF-κB) p65 activation. On the other hand, NF-κB p65 activation is a key regulator of the signal pathway that stimulates cell proliferation, growth, and migration (Li et al., 2014). Production of reactive oxygen species (ROS) or weakening of the antioxidant defense mechanism leads to DNA damage and stimulates the expression of NF-κB p65 (Janssens and Tschopp, 2006). In addition, NF-κB p65 is also the main regulator of oxidative stress response (Shu et al., 2014). Previous studies have shown that OA can cause oxidative stress in BRL-3A cells. However, it is not clear whether PPARγ and NF-κB p65 are activated and play an important role in this process.

Isoorientin (3′, 4′, 5, 7-tetrahydroxy-6-C-glucopyranosy flavone, ISO) is one of the effective active components extracted from humus and other plants. The molecular formula and molecular weight of ISO are C_21_H_20_O_11_ and 448.38, respectively. A previous study reported that isoorientin has anti-inflammatory, antiviral, and antioxidant effects (Fan et al., 2020). Moreover, isoorientin has a certain effect in relieving fat accumulation and clearance of ROS (Yuan et al., 2014; Mazibuko-Mbeje et al., 2020). Isoorientin can also inhibit MAPKs activation and NF-κB p65 nuclear translocation (Li Y. et al., 2020). In our previous study, we found that isoorientin can alleviate cadmium-induced injury of rat renal tubular epithelial cells and cell cycle arrest (Chen et al., 2020). However, it has not been reported whether isoorientin has a protective effect on hepatocyte steatosis induced by OA.

In this study, we used *in vivo* experiments in rats and combined with *in vitro* experiments in the rat hepatocyte cell line BRL-3A. We conducted Western blot analysis, flow cytometry, immunofluorescence, transmission electron microscopy, and immunohistochemical experiments to explore the role and mechanism of isoorientin in OA induced hepatocyte injury. Our findings will provide a theoretical basis for the clinical application of isoorientin.

## Material and Methods

### Chemicals and Antibodies

The BRL-3A cell line was obtained from the Cell Bank of the Institute of Biochemistry and Cell Biology (Shanghai, China). Dulbecco’s modified Eagle’s medium (DMEM), trypsin-EDTA, and fetal bovine serum (FBS) were purchased from Thermo Fisher Scientific (Waltham, MA United States), while Oleic acid (OA, O1383), isoorientin (ISO, I1536), and 2-(4-amidinophenyl)-1H-indole-6-carboxamidine (DAPI) were obtained from Sigma-Aldrich (St. Louis, MO, United States). Oil Red O stain kit was bought from Solabio Co., Ltd (Shanghai, China), and Reactive Oxygen (ROS) Detection Kits were obtained from Beyotime Biotechnology Co., Ltd (Shanghai, China). Annexin V-FITC apoptosis detection kits was purchased from BD Biosciences (San Diego, CA, United States). Horseradish peroxidase (HRP)-conjugated goat anti-rabbit immunoglobulin G was purchased from Santa Cruz Biotechnology (Santa Cruz, CA, United States). The TG assay kit (A110‐1‐1) was obtained from Nanjing Jiancheng Bioengineering Institute (Nanjing, China). Anti-β-actin (CST, 4970), anti-NF-κB (CST, 8242), and anti-Phospho-H2AX (CST, 9718) antibodies were obtained from Cell Signaling Technology Inc. (Danvers, MA, United States), while the anti‐HO-1 (Abcam; ab189491), anti-PPARγ (Abcam; ab272718), and anti-SREBP‐1 (Abcam; ab28481) antibodies were manufactured by Abcam Ltd (Cambridge, MA). Notably, the antibodies were diluted according to the manufacturer’s instructions. The other chemicals and reagents were purchased locally and were all of analytical grade.

### Cell Culture and Treatment

BRL-3A cells were cultured in DMEM supplemented with 10% FBS, followed by incubation at 37°C in a 5% CO_2_ atmosphere. When the cells covered the bottom of the cell culture bottle, they were digested using 0.25% trypsin for 0.5–1 min. Trypsin digestion was terminated with the medium containing serum. Next, the cells were resuscitated with fresh complete culture medium and inoculated into the new culture flask according to the proportion of 1:3, and the culture continued. Before treatment, we dissolved OA in anhydrous ethanol and the stock concentration was 0.6 M, while the stock concentration of ISO was 20 μM.

### Animals and Experimental Design

Thirty two female SD rats (body weight, 60–70 g) were acclimated for 1 week under well-controlled temperature and light conditions before the experiments were started. Rats were housed in polypropylene cages, with four rats in each cage. Next, the rats were randomly assigned into four groups: Group 1, control group (0.5 ml DDW); Group 2, OA group (0.5 ml OA); Group 3, OA + ISO group (0.5 ml OA+15 mg/kg ISO); and Group 4, ISO group (15 mg/kg ISO). OA and ISO were given by intragastric administration. Procedures involving the rats and their care were conducted in accordance with the Guidelines for Ethical Control and Supervision in the Care and Use of Animals. Moreover, the experimental procedures were approved by the (Permit Number: SYXK-2018-0010).

### Western Blot Analysis

After treatments, cellular proteins were extracted by ultrasonication in radio-immunoprecipitation assay lysis buffer, followed by determination of protein concentration using a BCA protein assay. Next, 40 μg protein per sample was resolved on a 6–15% SDS-PAGE gel and transferred onto a 0.22 μm PVDF or nitrocellulose membrane. Membranes were then blocked with 5% non-fat milk in TBST for 2 h at room temperature, followed by overnight incubation at 4°C with the relevant antibodies. Next, membranes were washed six times using TBST with shaking for 5 min, followed by incubation with HRP-conjugated secondary antibodies for 90 min at room temperature. After incubation with secondary antibodies, membranes were washed another six times with TBST. Western blots were developed using SuperSignal West Femto Chemiluminescent Substrate (ThermoFisher, United States). Finally, ImageJ software was used to quantify immunoreactive protein bands. It is worth noting that all assays were performed in triplicate.

### Evaluation of Cell Morphology and Ultrastructure Using Electron Microscopy

Scanning electron microscopy was used to evaluate cell morphology. Firstly, cells were seeded on sterile coverslips in 24-well plates. When the cells reached 60% confluence, they were treated with 0, 2.5, and 5.0 μmol/L Cd for 12 h, followed by washing twice with PBS and fixing in 2.5% glutaraldehyde solution overnight at 4°C. After fixation, cells were washed three times with PBS for 15 min, and then sequentially dehydrated with 50, 70, 80, 90, 95, and 100% ethanol. Samples were then dried at the CO_2_ critical point, and viscous samples were plated with gold. Finally, they were evaluated and photographed using a scanning electron microscope.

Observation of cell ultrastructure by transmission electron microscopy. After treatment with OA, cell samples were collected and fixed with 2.5% glutaraldehyde in 0.1 M PBS for 24 h. Then, samples were post-fixed in 1% osmium tetroxide fixative for 0.5 h at 4°C, washed three times with PBS and dehydrated in an ethanol series, sliced on an ultramicrotome to obtain ultrathin sections. Next, sections were dyed with lead citrate and uranyl acetate, followed observed using transmission electron microscopy (Tecnai 12, FEI, United States).

### Analysis of Intracellular ROS and Apoptosis Rate by Flow Cytometry

Detection of the reactive oxygen species (ROS) in BRL-3A cells was performed using the redox-sensitive dye, DCFH-DA. DCFH-DA was diluted 1:1000 with serum-free medium to a final concentration of 10 μmol/L. The diluted DCFH-DA was added to the collected cells, which were then incubated at 37°C for 20 min, with inversion mixing every 3–5 min. The cells were then washed three times with PBS to sufficiently remove the DCFH-DA that had not entered the cells. Next, the cells were filtered through a flow tube with a 200 mesh screen and the ROS content was measured using a flow cytometer.

To detect the apoptosis rate, cells were first seeded on sterile coverslips in six-well plates. Cells were cultured to 70% confluence and then they were treated with OA and/or ISO for 24 h. Next, cells were washed twice with PBS, and incubated with AnnexinV-FITC and PI for 15 min at room temperature in the dark. Cells were then washed twice with PBS, resuspended in 1 ml PBS, and observed by fluorescence microscopy. Notably, green fluorescence was only present in cells in early apoptosis, while cells in late apoptosis exhibited red fluorescence.

### Observation of Lipid Droplet Production by Oil Red O Staining

Firstly, we inoculated BRL-3A cells on the six-hole plate. When the cells were cultured to the logarithmic growth phase, they were grouped and treated according to the experimental design. We then discarded the culture medium and washed the cells twice with 1 × PBS. Next, the cells were stained with Oil Red O for 15 min and the stained sections were washed using distilled water at a temperature of 37°C. The sections were then counterstained with Mayer’s hematoxylin for 4 min, followed by washing for 2 min with distilled water. Finally, the sections were mounted in aqueous mountant and observed under a fluorescence microscope (Leica 2500; Leica Corporation, Germany).

### Detection of Intracellular Triglyceride Content

Cells were lysed to prepare the cell homogenate. We then directly determined the content of the protein and the level of triglyceride (TG) in the lysate using BCA and TG kits, respectively, through a microplate reader. Finally, the ratio of the lipid level to the protein concentration was calculated to represent the relative content of intracellular TG.

### Immunofluorescence Assay

BRL-3A cells were fixed with 4% paraformaldehyde for 30 min and permeabilized with 0.5% Triton-100 for 15 min at room temperature. This was followed by blocking in 5% bovine serum albumin for 2 h, and overnight incubation with diluted primary antibody at 4°C. After washing twice with PBS, cells were incubated with secondary antibodies for 1 h at room temperature. They were again washed twice with PBS, incubated with 5 μg/ml TRITC-phalloidin for 30 min in the dark, and then washed three times with PBS. Finally, the cells were stained with 100 μg/ml DAPI for 15 min, washed twice with PBS, and then visualized under a confocal fluorescence microscope (Leica, Wetzlar, Germany).

### Observation of Histological Changes of Liver by Hematoxylin and Eosin Staining

Liver tissues were cut into small pieces of 0.2–0.5 cm and soaked in 10% neutral formalin solution. We then changed the fixed fluid after 24 h to remove the excess blood. Next, we conducted routine dehydration, wax dipping, embedding, slicing, and H&E staining. Finally, the specimens were observed and photographed under a light microscope.

### Immunohistochemistry

The paraffin slices were put into xylene I, II, III for 10 min, followed by sequential dehydration in ethanol gradient (high to low: 100, 95, 80, and 70%) for 2 min. Next, the slices were washed three times with PBS for 5 min each time. We then soaked the slices with preheated 0.3% Triton X100 under dark conditions for 30 min and washed them three times with PBS for 5 min each time. Next, we added the diluted primary antibody and stored at 4°C for 24 h. After the antibody was absorbed, we washed three times with PBS for 5 min each time. We then added the diluted secondary antibody and incubated at room temperature for 2 h. Next, we washed with PBS three times for 5 min each time. This was followed by addition of DAB for 10 min, and washing three times with PBS for 5 min each time. We then added chromogenic solution and performed immunohistochemical analysis for 30 min. Finally, we rinsed the colored film with clean water for 30 s and then soaked the slices in hematoxylin for staining. After dehydrating the gradient alcohol, we sealed the film and captured the images using a fluorescence microscope.

### Statistical Analysis

Data were analyzed by one-way analysis of variance (ANOVA) using SPSS 22.0 software and presented as mean ± SD. Student’s *t*-test was used to determine statistical significance between two groups. Notably, differences were considered to be statistically significant at *p* < 0.05 and highly significant at *p* < 0.01.

## Results

### Isoorientin can Alleviate Oleic Acid-Induced Hepatotoxic Injury and Steatosis in Rat Liver

**FIGURE 1:**
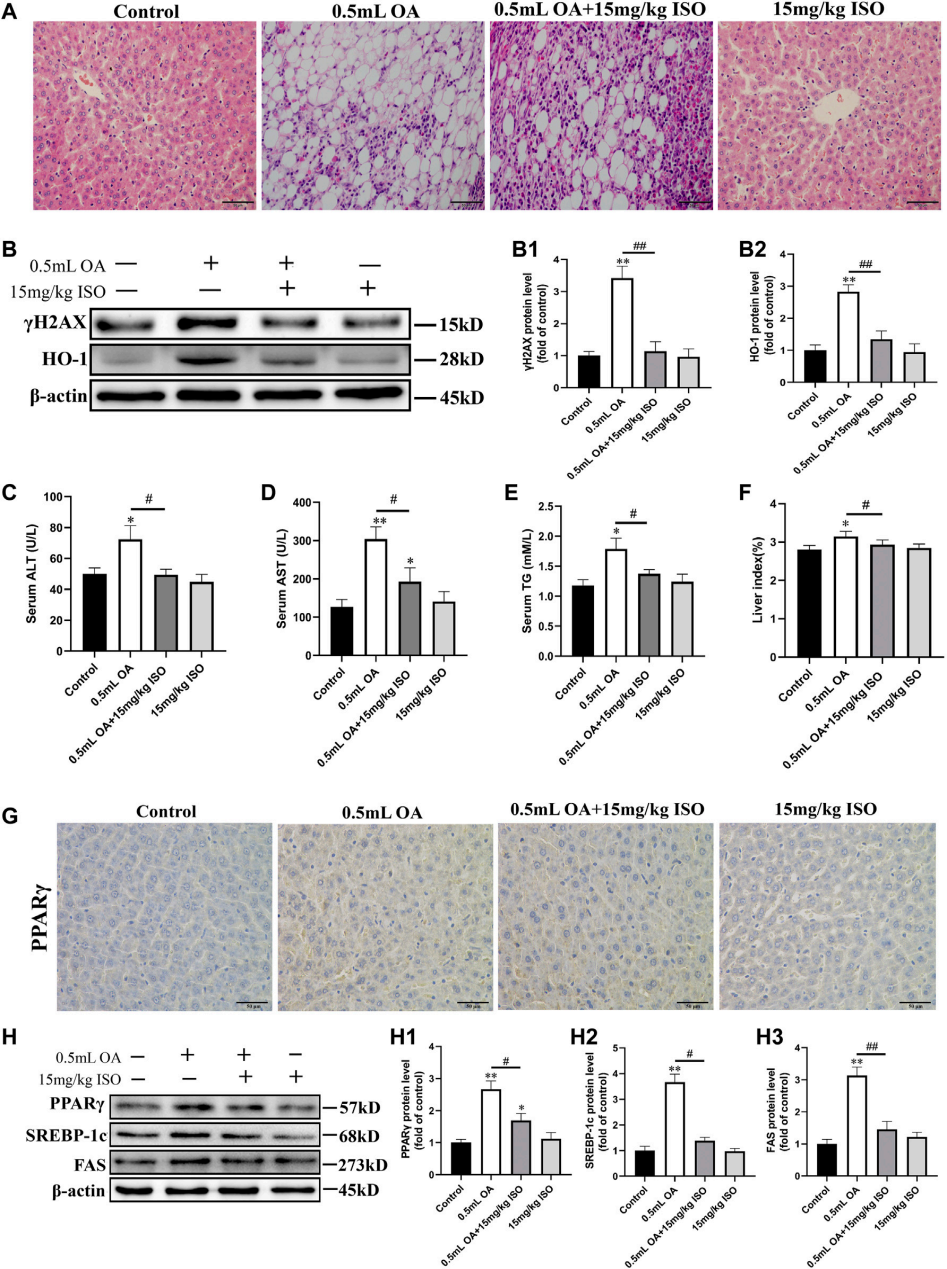
The role of isoorientin in oleic acid-induced hepatotoxicity in rats. **(A)** Observation of pathological changes of liver tissue by H&E staining (scale bar = 50 μm). **(B)** Determination of γH2AX and HO-1 protein expression in liver tissue using Western blot analysis (*n* = 3). **(C–E)** Biochemical detection of ALT, AST, and TG in serum (*n* = 3). **(F)** Statistical results of liver index in rats (liver index refers to the ratio of the weight of the liver to the body weight of the rat). **(G)** The expression of PPARγ protein in liver tissue was observed by immunohistochemistry (scale bar = 50 μm). **(H)** Western blot analysis was used to determine the expression of PPARγ, SREBP-1c, and FAS protein in liver tissue (*n* = 3). All results are expressed as the mean ± SD. Compared to the control group, ^*^ *p* < 0.05, ^**^ *p* < 0.01. Comparison between the two groups, ^*#*^ *p* < 0.05, ^##^ *p* < 0.01.

We observed the pathological changes of liver tissues using pathological sections, and the obtained results are shown in Figure 1A. Results indicated that the liver lobules in the control group had clear structures, the liver cells were polygonal with clear boundaries, and no lipid droplet infiltration was found. In the OA treated group, the structural boundaries of the hepatic lobules were not clear, and the hepatocytes were filled with lipid droplets of different sizes. Therefore, the cytoplasm of the hepatocytes was vacuolated and a large number of adipocytes and inflammatory cells could be seen. In addition, the morphology of hepatocytes was improved, and the number of lipid droplets and vacuolation were decreased in the OA and ISO co-treatment group compared to the OA alone treatment group. There was no visible lesions in the liver of the isoorientin group.

Western blot analysis results showed that the expression of γH2AX and HO-1 protein in liver of the OA treated group were significantly higher than in the control group. After co-treatment with isoorientin, the expression of γH2AX and HO-1 protein were significantly lower than in the OA treated group (Figure 1B). The contents of ALT, AST, and TG in the serum of rats in the OA treatment group were significantly increased, while the contents of ALT, AST, and TG were significantly lower in the OA co-treatment with isoorientin group than those in the OA treatment group. There was no significant difference between the isoorientin treatment group and the control group (Figures 1C–E). In addition, the results of organ index showed that the liver index of rats treated with OA increased significantly, but the liver index was significantly lower in the OA and ISO co-treatment group than in the OA treatment group (Figure 1F).

We used immunohistochemistry to detect the expression of PPARγ in liver tissues, with results (Figure 1G) showing that the brown-yellow in the OA treatment group increased significantly. Besides, the isoorientin and OA co-treatment can significantly reduce the degree of brown-yellowness. There was no significant difference between the isoorientin group and the control group. Furthermore, we used Western blot analysis to detect the expression of fat synthesis-related proteins in liver tissues. Results (Figure 1H) showed that the expression of PPARγ, SREBP-1c, and FAS proteins in the OA group were significantly higher than in the control group. The expression of PPARγ, SREBP-1c, and FAS protein decreased significantly in the isoorientin co-treatment with OA group compared to the OA group. However, there was no significant difference in the expression of these proteins between the isoorientin group and the control group.

### Oleic Acid Induced Oxidative Damage of BRL-3A Cells

**FIGURE 2:**
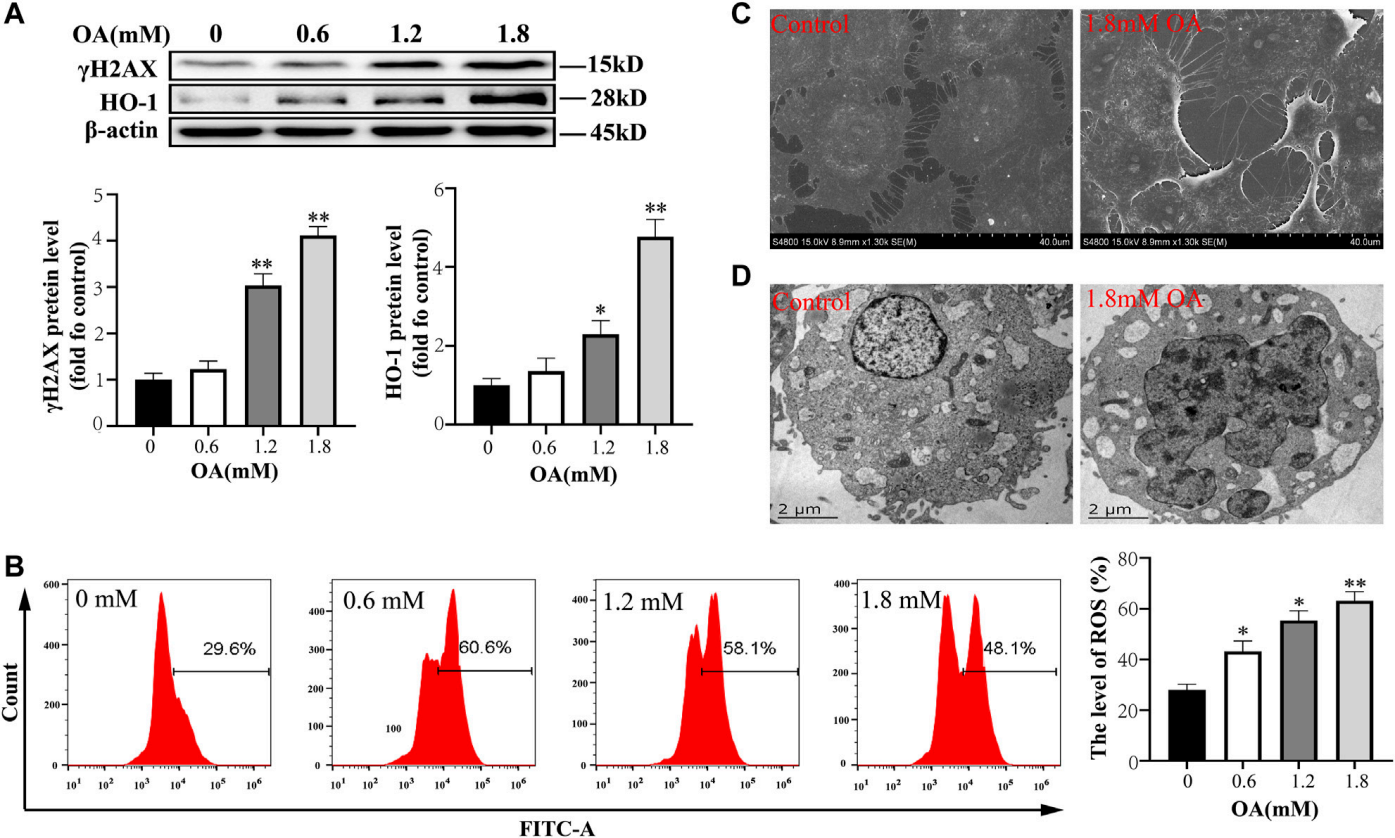
Oleic acid causes cytotoxic damage. After BRL-3A cells were treated with OA for 24 h, **(A)** we determined the expression of oxidative damage related proteins using Western blot analysis (*n* = 3). **(B)**The content of intracellular ROS was detected by flow cytometry (*n* = 3). **(C, D)** The ultrastructural changes of cells were observed under a scanning electron microscope and transmission electron microscope. All results are expressed as the mean ± SD. Compared to the control group, ^*^ *p* < 0.05, ^**^ *p* < 0.01.

In *in vitro* experiments, we first detected the oxidative damage of OA to cells, and the results showed that the expression of γH2AX and HO-1 protein, and the concentration of intracellular ROS increased with increase of OA concentration and there were significant differences in the concentration of 1.2 mM OA compared to the control group (Figures 2A,B). In addition, electron microscopy showed that the cytoskeleton was destroyed after OA treatment, and the number of microtubule microfilaments decreased, they were broken or even disappeared in some instances (Figure 2C). The nucleus was pyknotic, dissolved, and deformed, and a large number of vacuoles appeared in the cells (Figure 2D). These results suggest that OA treatment leads to oxidative damage of cells.

### Oleic Acid Induced Cellular Steatosis

**FIGURE 3:**
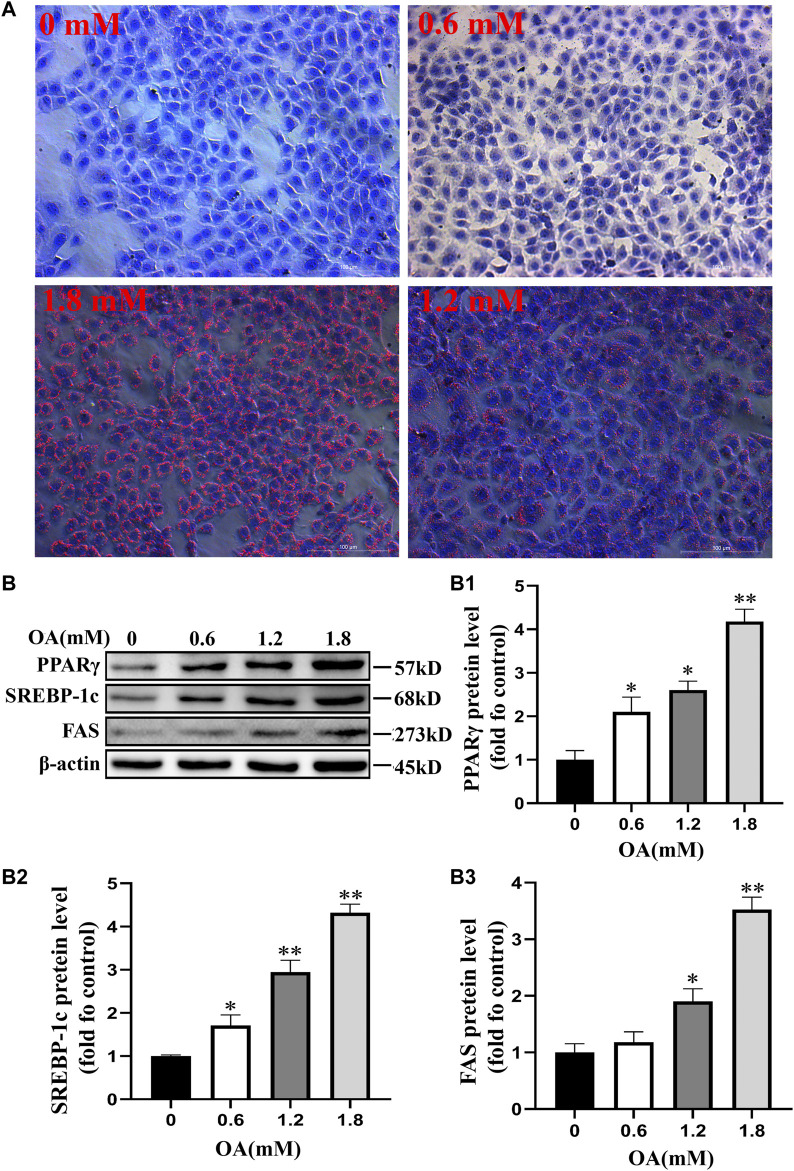
Steatosis of BRL-3A cells induced by oleic acid. After the cells were treated with OA for 24 h, **(A)** the content of lipid droplets was observed by oil red O staining (scale bar = 100 μm). **(B)** Detection of the expression of proteins associated with fat synthesis through Western blot analysis (*n* = 3). All results are expressed as the mean ± SD. Compared to the control group, ^*^ *p* < 0.05, ^**^ *p* < 0.01.

We used oil red O staining to observe the effect of OA treatment on lipid droplet production in BRL-3A cells. The results showed that production of the intracellular lipid droplet increased significantly with the increase of OA concentration compared to the control group (Figure 3A). Western blot analysis results (Figure 3B) also showed that the expression of PPARγ, SREBP-1c, and FAS protein increased significantly with the increase of OA concentration (*p* < 0.05 or *p* < 0.01). These results suggest that OA treatment induces steatosis in BRL-3A cells.

### Isoorientin can Alleviate the Oxidative Damage Induced by Oleic Acid in BRL-3A Cells

**FIGURE 4:**
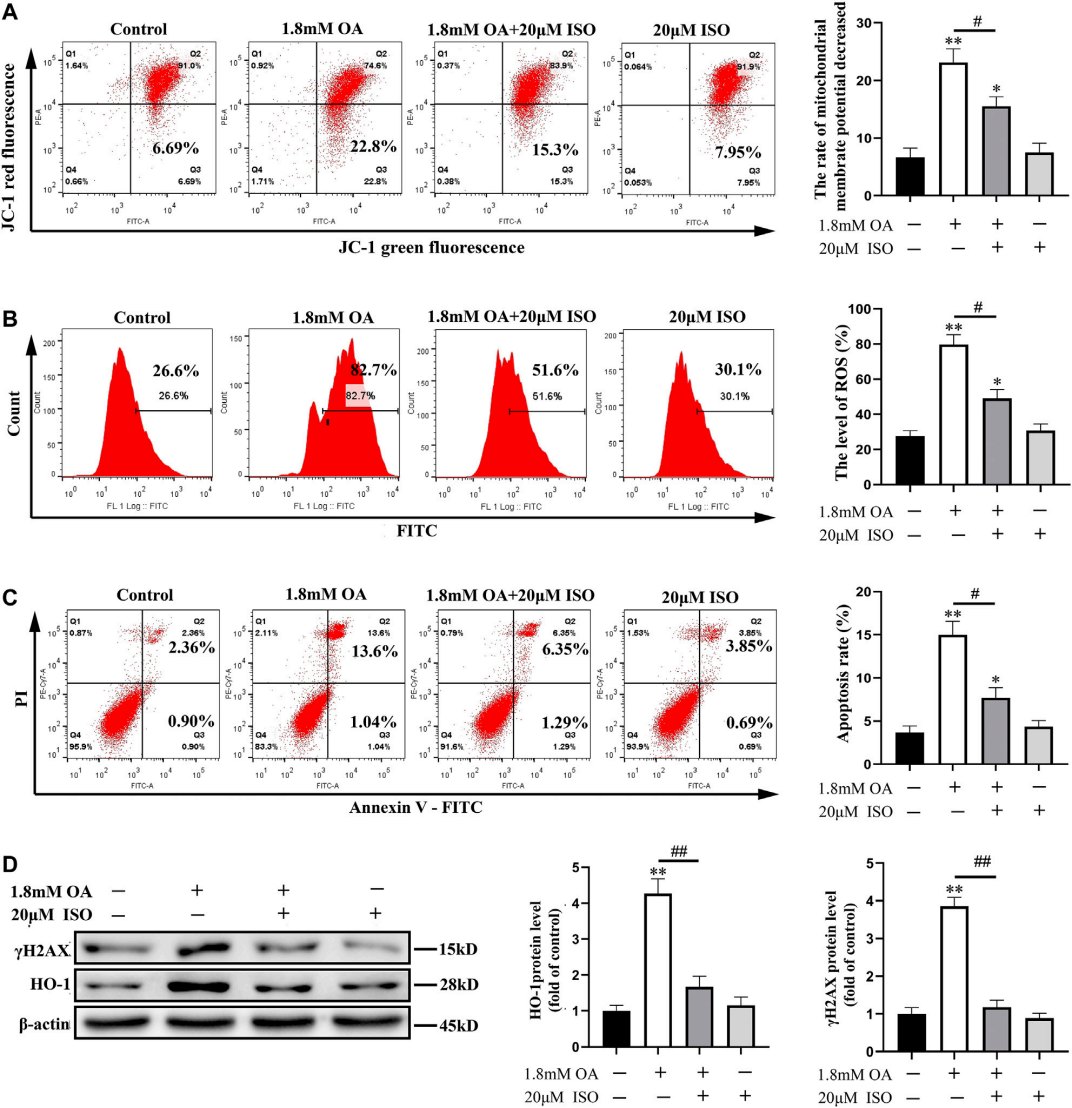
Isoorientin can reduce the oxidative damage of cells induced by oleic acid. After the cells were co-treated with isoorientin and OA for 24 h, the mitochondrial membrane potential **(A)**, the content of ROS **(B)**, and the rate of apoptosis **(C)** were measured using flow cytometry. **(D)** Western blot analysis was used to detect the expression of γ H2AX and HO-1 protein (*n* = 3). Compared to the control group, ^*^ *p* < 0.05, ^**^ *p* < 0.01. All results are expressed as the mean ± SD. Comparison between the two groups, ^*#*^ *p* < 0.05, ^##^ *p* < 0.01.

Furthermore, we conducted an experiment to determine whether isoorientin, an antioxidant, can reverse the oxidative damage in OA-treated cells. Results showed that the mitochondrial membrane potential of OA-treated cells decreased significantly compared to the control group, while the content of ROS and the rate of apoptosis increased significantly in OA-treated cells compared to the control group. Moreover, the mitochondrial membrane potential increased significantly, while the content of ROS and the rate of apoptosis decreased significantly in ISO-treated cells compared to the oleic acid group. The expression of γ H2AX and HO-1 were also significantly decreased (Figure 4) in the isoorientin and oleic acid co-treatment group. These results suggest that isoorientin can alleviate the oxidative damage induced by OA and inhibit its induced apoptosis.

### Isoorientin Alleviates Oleic Acid-Induced Steatosis by Regulating the PPARγ/NF- κB p65 Signal Pathway

**FIGURE 5:**
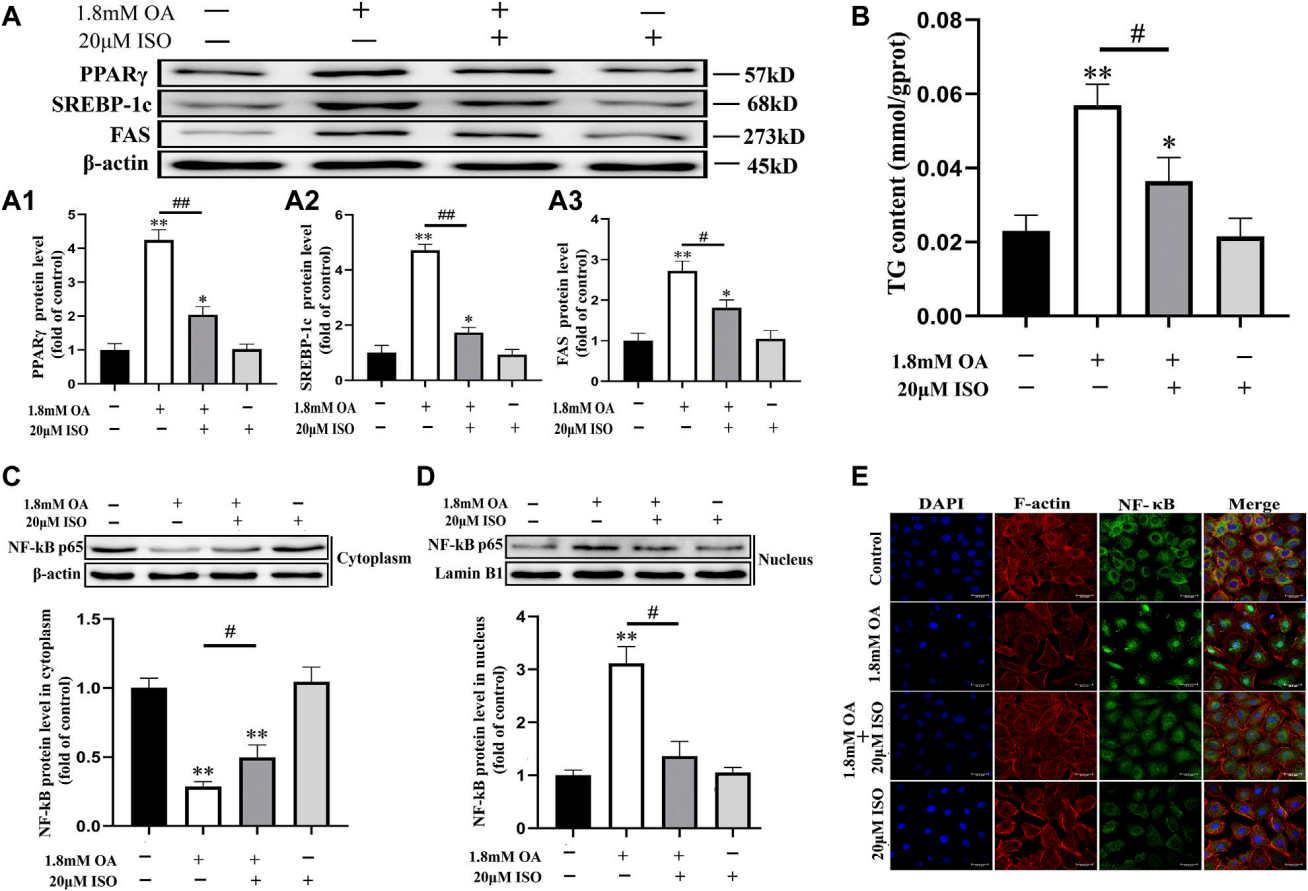
The role of the PPARγ/NF- κ B p65 signal pathway in the antagonistic effect of isoorientin on oleic acid-induced cellular steatosis**.** The cells were co-treated with isoorientin and OA for 24 h, **(A)** the expression of PPARγ, SREBP-1c, and FAS protein was determined using Western blot analysis (*n* = 3). **(B)** The content of TG in the cells was detected using the detection kit (*n* = 3). **(C, D)** The cytoplasm and nuclear proteins were extracted and the nuclear translocation of NF-κB p65 protein was detected (*n* = 3). **(E)** Immunofluorescence was used to observe the changes of the cytoskeleton and the distribution of NF-κB p65 (scale bar = 30 μm). All results are expressed as the mean ± SD. Compared to the control group, ^*^ *p* < 0.05, ^**^ *p* < 0.01. Comparison between the two groups, ^*#*^ *p* < 0.05, ^##^ *p* < 0.01.

We used Western blot analysis to determine the expression of PPARγ, SREBP-1c, and FAS proteins, and the nuclear transcription of NF-κB p65. Results showed that the content of NF-κB p65 decreased significantly in the cytoplasm, while the content increased significantly in the nucleus after OA treatment. The contents of PPARγ, SREBP-1c, FAS, and NF-κB p65 protein were significantly decreased in the nucleus, while the content of NF-κB in the cytoplasm was significantly increased in the isoorientin and OA co-treatment group compared to the OA alone treatment group (Figures 5A–D). Immunofluorescence results also showed that OA treatment led to significant nuclear translocation of NF-κB p65, while isoorientin intervention could inhibit OA-induced nuclear translocation (Figure 5E). In addition, the content of TG increased significantly after OA treatment, but decreased significantly after the intervention of isoorientin. These results indicate that the PPARγ/NF-κB p65 signaling pathway plays an important role in isoorientin alleviating fatty degeneration and oxidative damage caused by OA treatment.

## Discussion

Previous studies have shown that OA can induce steatosis and injury of rat hepatocytes, and the effect of isoorientin on antioxidation has also been confirmed (Zhang et al., 2019; Chen et al., 2020). The main aim of this study was to reveal the role of isoorientin in OA-induced hepatocyte injury and steatosis in rats. Our results showed that isoorientin can reduce the oxidative damage and steatosis of hepatocytes induced by OA, with activation of the PPARγ/NF-kB p65 pathway playing a vital role.

Alanine aminotransferase (ALT) is mainly found in hepatocytes, while aspartate aminotransferase (AST) mainly exists in the myocardium, followed by liver, kidney, and other tissues (Smith et al., 2020). Generally, the content of serum AST is low, but AST will be released into the blood when cells are injured, thus, AST is often used as a serum enzyme index for the diagnosis of liver injury (Vonbank et al., 2015; Li, 2020). Determination of serum ALT, AST, TG, and other indexes is very important for evaluation of nonalcoholic fatty liver. In this study, we used *in vitro* rat experiments to verify the role of isoorientin in cellular oxidative damage and steatosis induced by OA. Results showed that the liver tissue of the rats in the OA group had obvious lipid vacuolation, and the serum ALT, AST, and TG contents increased significantly. Furthermore, the expression of γH2AX, HO1, PPARγ, and SREBP-1c increased significantly in liver tissues, suggesting that OA treatment leads to oxidative damage and steatosis in rat liver. However, the number of lipid droplets and vacuolation in liver tissues decreased significantly after co-treatment of isoorientin with OA, and the contents of ALT, AST, and TG in serum also decreased significantly. In addition, the expression of γH2AX, HO-1, PPARγ, and SREBP-1c protein decreased significantly. Collectively, these results showed that isoorientin alleviated fatty degeneration and oxidative damage of liver tissue induced by OA.

OA is an essential unsaturated fatty acid for the body and it is reported that it has anti-oxidation and hypolipidemic effects at a suitable concentration. However, OA can induce oxidative damage to cells in excessive amounts, which is also confirmed by the results of this study.

The liver is the main fat storage organ, which is associated with hypertension, hyperlipidemia, diabetes, and other diseases (Moreno-Fernandez et al., 2021; Parente et al., 2021). Among liver diseases, fatty liver disease has a very high incidence, and fatty liver disease can cause serious consequences such as liver cirrhosis and liver cancer (Powell et al., 2021). Previous studies have reported that OA can induce steatosis in a variety of cells, thus, it is often used as an inducer for *in vitro* models of non-alcoholic fatty liver (Khoo et al., 2019; Rafiei et al., 2019). Moreover, the content of intracellular ROS and HO-1 protein is the key index of oxidative stress (Joshi et al., 2021), and the expression of γH2AX is an important indicator for detecting DNA damage (Singh et al., 2015; Collins et al., 2020). Previous experimental results showed that OA can cause toxic damage and steatosis in BRL-3A cells, while results obtained in this study have shown that OA treatment significantly increased the content of ROS, and the expression of HO-1 and γH2AX proteins. In addition, OA caused oxidative damage and increased the apoptosis rate. However, co-treatment of isoorientin with OA could significantly reduce the oxidative damage and apoptosis rate induced by OA, and the mechanism may be associated with the antioxidation effect of isoorientin.

It is worth noting that there is a correlation between oxidative stress and lipid metabolism. Lipid metabolic disorders often produce ROS, which leads to oxidative damage. Studies have also shown that ROS can regulate lipid metabolism (Albaghdadi et al., 2021; Chen et al., 2021; Li et al., 2021). Aberrant metabolism of TG is the main cause of fatty liver, and the most intuitive manifestation in steatosis is the accumulation of TG (Yu et al., 2021; Zhang Z. et al., 2021). Therefore, we determined the content of TG in this study, with results showing that OA treatment significantly increased the content of TG both *in vivo* and *in vitro*. On the other hand, isoorientin treatment could reduce the content of TG increased by OA treatment. Notably, PPARγ is closely associated with fat formation and development, and is highly expressed in adipose tissues (Xie et al., 2020). In addition, PPARγ modulates lipid metabolism, and participates in the regulation of fatty acid release, transport and storage, and other related genes (Mwangi et al., 2016). PPARγ can regulate the expression of fatty acid release, transport and storage genes, such as lipoprotein lipase and fatty acid transporter CD36. Even PPARγ can induce the transformation of myoblasts into adipocytes, thus playing an important role in lipid metabolism. SREBP-1c is an important nuclear transcription factor in animal fat metabolism and it regulates the synthesis of animal fat by regulating the gene expression of enzymes associated with fat metabolism such as FASN (Zhang W. et al., 2021). SREBP-1c also participates in the synthesis and uptake of cholesterol, fatty acids, and triglycerides. Studies have shown that overexpression of SREBP-1c can lead to the disorder of lipid metabolism and increase production of lipids (Luan et al., 2020; Zhang et al., 2020). NF- κ B p65 is the main regulator of acquired immune response. After entering the nucleus, NF- κ B p65 binds to the gene with NF- κ B binding site and initiates the transcriptional process. In addition, NF-κB p65 protein induces migration and invasion by inhibiting apoptosis and promoting cell proliferation (Taniguchi and Karin, 2018; Hacioglu et al., 2021).

Previous studies have reported that PPARγ can alleviate lung injury by inhibiting activation of NF-κB (Lin et al., 2015), and the results of this study on liver cells showed that the expression of PPARγ, SREBP-1c, and FAS was increased after OA treatment, but the expression of these proteins were significantly decreased after co-treatment of isoorientin with OA. Moreover, we detected the content of NF-κB in the cytoplasm and nucleus. Obtained results showed that OA treatment promoted the nuclear translocation of NF-κB p65, indicating that NF-κB p65 was activated. In addition, isoorientin significantly inhibited OA-induced NF- κB p65 nuclear translocation, suggesting that isoorientin may play a regulatory role through the PPARγ/NF- κB p65 pathway. However, the conclusions drawn from experiments on female rat liver and rat liver cell lines in this study have certain limitations. Later studies need to be carried out on more species and cells to further confirm the conclusions of this article.

## Conclusion

**FIGURE 6:**
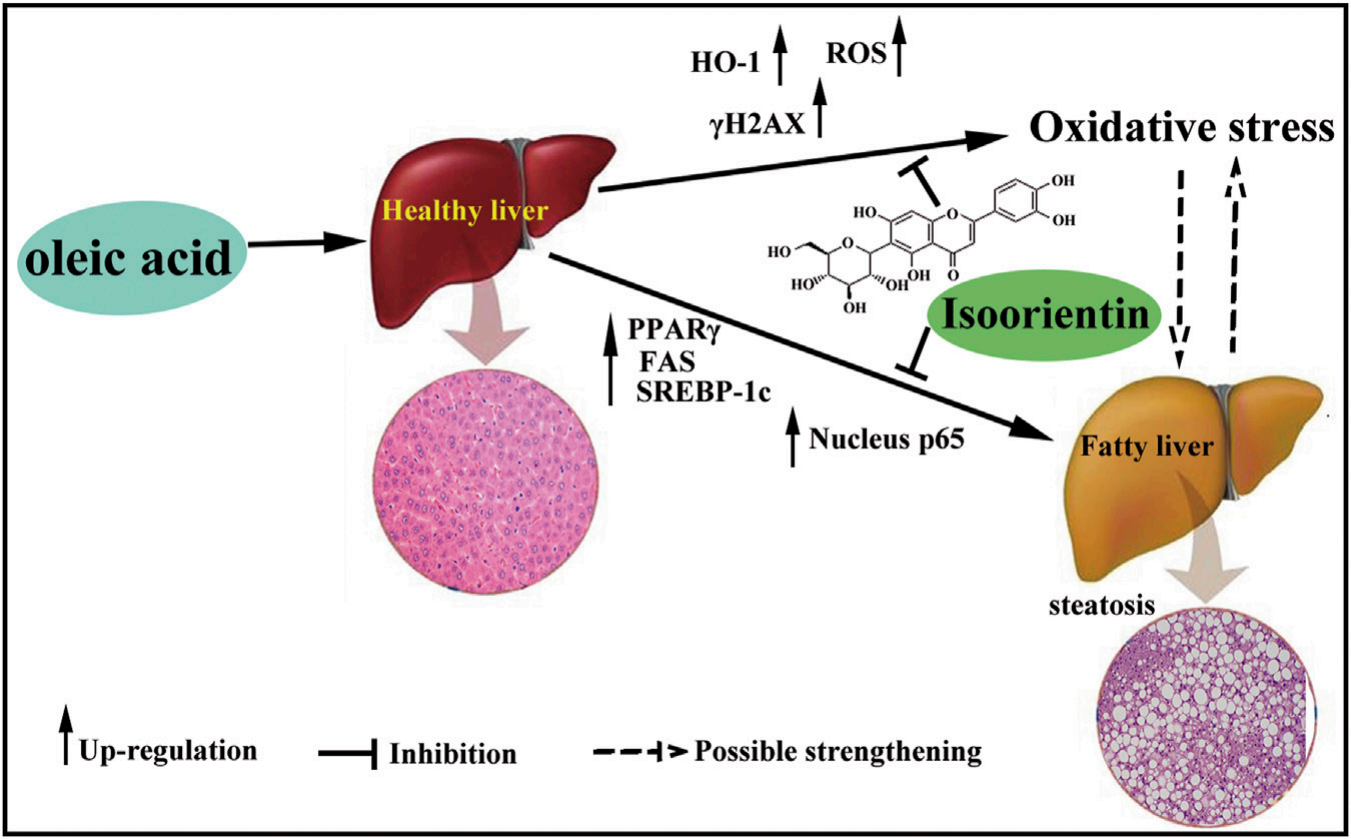
Schematic diagram of the role of isoorientin in oleic acid-induced liver cell damage. OA treatment induce oxidative damage and steatosis in rat liver cells, and isoorientin can significantly inhibit OA-induced oxidative damage and steatosis *via* PPARγ/NF-κB p65 signal pathway.

OA treatment can induce oxidative damage and steatosis in rat liver cells, and isoorientin can significantly inhibit OA-induced oxidative damage and steatosis. Furthermore, the PPARγ/NF-κ B p65 signal pathway plays an important regulatory role in this process (Figure 6). Our results will provide a theoretical basis for the treatment of nonalcoholic fatty liver disease and expand the clinical application of isoorientin.

## Data Availability

The original contributions presented in the study are included in the article/Supplementary Material, further inquiries can be directed to the corresponding authors.
